# Plants’ healthiness assessment as part of the environmental monitoring of protected mountainous area in the example of Karkonosze (Giant) Mts. (SW Poland)

**DOI:** 10.1007/s10661-016-5551-5

**Published:** 2016-09-02

**Authors:** Wojciech Pusz

**Affiliations:** Department of Plant Protection, Wroclaw University and Environmental and Life Sciences, Grunwaldzki Sq. 24A, 50-363 Wroclaw, Poland

**Keywords:** Plant diseases, Fungal diseases, Pathogenic fungi, Environmental monitoring

## Abstract

The aim of phytopathological monitoring is to check the healthiness of plants and observe the changes that occur in their populations. In the vast majority, these types of observations are conducted in agriculture and forestry. An interesting aspect of phytopathological monitoring is the assessment of the origin of the plant species. The research of fungal communities (including pathogens) in plants may, for example, indicate the relic nature of the plant species. Reduction of the occurrence or disappearance of fungi species associated with its host plant can evidence slow decline of their habitats. This applies mainly to arctic-alpine fungal species. On the other hand, for some plant hosts, colonization of their organs by polyphagous fungi is being recorded. One such example is the downy willow, on which six species of fungi were found in the Karkonosze Mts. In 2014, there were no fungi found on this plant. However, comparing the species composition of fungi associated with downy willow given by Schroeter (1908) to the contemporary one and to the study results of other researchers, a decrease in the number of fungi species is clearly visible. This may be related to the environmental pollution, which took place in the Sudetes in the second half of the twentieth century. For instance, the species of the genus *Rhytisma* colonize the leaves of trees and shrubs and are particularly sensitive to the concentration of SO_2_ in the air, but nobody has looked for this fungus on this host in the past. Yet, presently, we were able to find *Rhytisma* fungus in Karkonosze Mts. Phytopathological monitoring was conducted in the years of 2014–2015 in the sub-alpine zone of the Karkonosze (Giant) Mts. It has been shown that, compared to similar studies conducted in the 1990s of the twentieth century, the species composition of fungi infesting *Rubus chamaemorus* and *Betula carpactica* has changed. Is this the beginning of changes that will occur in populations of plants?

## Introduction

One of the most advanced environmental monitoring systems in Poland is the “*Integrated Monitoring of Natural Environment*” (IMNE), which is part of the State Environmental Monitoring. The goal of IMNE is to provide data about the representative ecosystems of Poland, taking into account both the evaluation components relevant to the general condition of the environment, as well as those related to its biodiversity. It is supposed to broaden the knowledge of the mechanisms of the ecosystem functioning, as well as the trends of changes taking place due to human activity, climate change and the impact of other factors on the individual elements of an ecosystem. One of the main tasks of IMNE is forest monitoring. The actions concerning monitoring of forest areas are described by the Polish Environmental Protection Law and are included in the regulations of various internal normative institutions established to protect nature (e.g. national parks or nature reserves). Despite the very extensive research related to environmental monitoring, including interalia studies of soil chemistry, meteorological measurements or research of the chemical composition of the assimilation apparatus of trees, the continuous phytopathological monitoring is rarely added to the list of these studies. Such attempts were made in Karkonosze National Park, where, for instance, at the beginning of the XXI century, the health status of dwarf mountain pine had been assessed (Pusz et al. 2013). However, in other mountainous areas covered by active measures of nature protection, such studies have not been conducted.

The prelude to phytopathological monitoring is undoubtedly an accurate recognition of the species composition of plant pathogens in a given area. The first attempt at a complex study of the species composition of fungi in Karkonosze took place in the late nineteenth century. It was conducted by Schroeter (1908), who listed in his monograph nearly 325 species of fungi. Most of them were either species associated with the soil substrate or common plant pathogens, such as representatives of *Erysiphales*, *Uredinales* and *Ustilaginales* or more broadly of *Pyrenomycetes*, *Discomycetes* and *Heliotiales* (these categories are old and now is not used by taxonomists). In his monograph, Schroeter lists, among others, quite rare rusts, such as *Coleosporium sonchi* and *Uromyces cacaliae* on *Adenostyles alliariae*. Also, at the end of the nineteenth century, a similar study started in Tatra Mountains (the highest mountain range in Poland). Works of Krupa (1886, 1888) and Raciborski (1887) laid the foundations for further study of fungi—particularly the pathogenic fungi of plants.

An interesting aspect of phytopathological monitoring is the assessment of the origin of the plant species. The research of fungi communities (including pathogens) in plants may, for example, indicate the relic nature of the plant species. Chlebicki (1997) studied the relic status of the bog sedge (*Carex magellanica*) and highland rush (*Juncus trifidus*) by analysing the composition of fungi species present in these plants and comparing them with the populations found in other regions of Poland and Europe. During the research on the bog sedge, the author observed six species of fungi, among which alpine species were recorded: *Arthrinium puccinioides* and *Phaeosphaeria alpina*, as well as arctic-alpine species such as *Coronellaria caricinella*. The presence of these species clearly demonstrates the relic origin of the Karkonosze bog sedge population. Among other plants growing in Karkonosze, where the species composition of local fungal communities was analysed, plants such as cloudberry (*Rubus chamaemorus*), *Betula pubescens* ssp. *Carpatica*, fernweed (*Pedicularis sudetica* spp. *sudetica*), snow saxifrage (*Saxifraga nivalis*) and downy willow (*Salix lapponum*) can be distinguished.

A decline of incidence or complete disappearance of fungi species tightly associated with their host plant can be the evidence for the slow decline of their habitats. This applies mainly to arctic-alpine fungal species. On the other hand, for some plant hosts, colonization of their organs by polyphagous fungi is being recorded. One such example is the downy willow, on which the six species of fungi were found on twigs in Karkonosze (Chlebicki 1999).

However, when comparing the species composition of fungi associated with downy willow given by Schroeter (1908) with the present one and with the study results of other researchers, a decrease in the number of species of fungi is clearly visible. This may be related to the environmental pollution, which took place in the Sudetes in the second half of the twentieth century. For example, the species of the genus *Rhytisma* (Chlebicki 1999), which colonize the leaves of trees and shrubs and are particularly sensitive to the concentration of SO_2_ in the air, were nowhere to be found previously. Presently, the authors were able to find *Rhytisma* fungus in Karkonosze Mts.

The goal of the present study was to determine the health status of rare and endangered species of plants found within the Karkonoski National Park (further abbreviated as KNP). The study also attempted to find out whether the phytopathological monitoring may become useful and successfully planted within the framework of natural environment monitoring systems, with particular attention paid to monitoring systems within protected areas.

## Materials and methods

### Study site

Karkonosze (Giant) Mts. are located in the Central Europe in the borderland between Poland and the Czech Republic. They are characterized by varying geological formation, varied land relief, severe alpine climate and crude vegetation cover compared to other European mountain ranges. In Karkonosze (Giant) Mts., there are altitudinal zonations: subalpine zone 1250–1450 m above sea level is covered with mountain pine (*Pinus mugo* Turra), the endemic and relict species in those mountains (Przewoźnik 2008).

The Karkonosze Mts. are a relatively low mountain range raised during the Alpine orogeny, with its highest summit, Śnieżka, amounting to merely 1602 m AMSL. On the other hand, it is an old and eroded range (Szczęśniak et al. 2009). The latter fact, among other factors, fostered creation of the high-altitude enclaves characterized by the high degree of rockiness and formation of post-glacial cirques, in which rare and very rare plant species occur that are classified as glacial relics and endemites (Chlebicki 2002; Szczęśniak et al. 2009; Dworzycki and Kroczek 2013).

During the last decades, a reduction in population counts of rare plant species has been observed in Karkonosze Mts. In extreme situations, such reductions may cause the population to die out (Żołnierz et al. 2004). Unfortunately, the endangered plant species are those which signify the exceptional character of Karkonosze Mts. These include endemites such as *Campanula bohemica* and glacial relics *Allium sibiricum*, *Arabis alpina* and *Geum montanum*, as well as the species of the genus *Saxifraga*, *Saxifraga nivalis* and *Saxifraga moschata* (Mirek et al. 2002; Żołnierz et al. 2004; Szczęśniak et al. 2009). That picture becomes even grimmer when contemporary floristic analyses are compared to historical ones, showing considerable reduction in species number over the time span considered (Fabiszewski and Kwiatkowski 2002).

### Data collection, sampling points and procedure of healthiness analysis

**Fig. 1:**
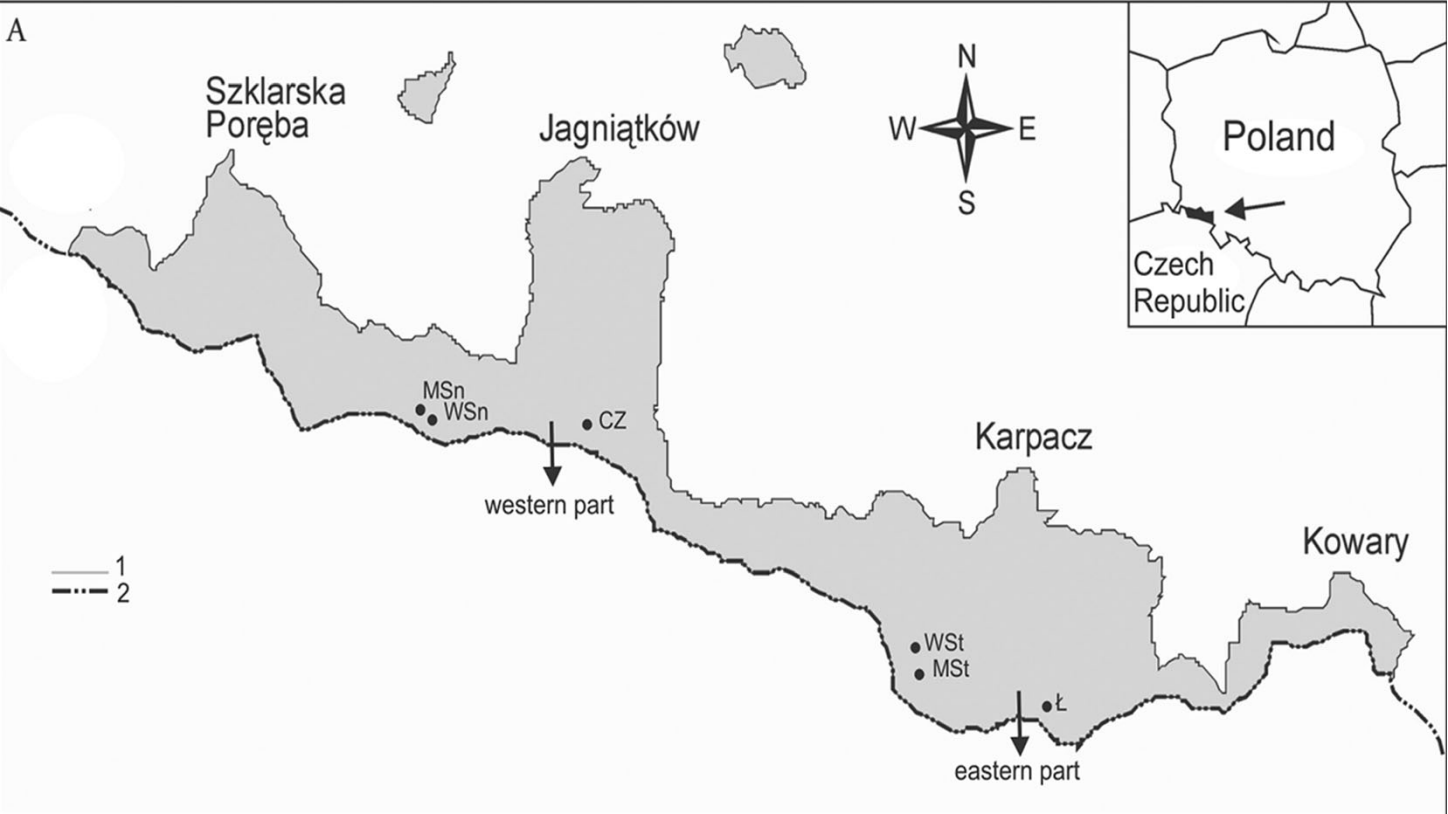
General view of the locations of the studied glacial cirques in the Karkonosze Mts. range and detailed maps of their situation in the western part and eastern part of the range Explanations: *CZ* Czarny Kocioł, *Ł* Kocioł Łomniczki, *MSn* Mały Śnieżny Kocioł, *MSt* Kocioł Małego Stawu, *WSn* Wielki Śnieżny Kocioł, *WSt* Kocioł Wielkiego Stawu; *1* borders of Karkonosze National Park, *2* state borders; (prepared by Ewa Fudali 2012)

Altogether, 55 plant species were encompassed by the healthiness monitoring conducted, and these included trees, shrubs and annual plants classified in different IUCN categories (Table 1). The observations were carried in the Live Gene Bank of KNP in Jagniątków (further abbreviated as LGB) and in the selected natural sites located within the KNP area, i.e. in the glacial cirques Kocioł Łomniczki, Mały Śnieżny Kocioł, Wielki Śnieżny Kocioł and Czarny Kocioł Jagniątkowski, as well as at Sokolnik, Petrovka and Chojnik—see Fig. 1. The observations were carried out in 3-week intervals during the growth seasons from 2014 to 2015. The plant health status was assessed in 21 plant species in the LGB but in as many as 45 plant species in the natural locations. In the course of the field observations, the disease symptoms were determined and the degree of infestation estimated. The degree of infestation was expressed in percentage of the plant surface area covered by disease symptoms (disease index) and as percentage of the number of individual plants infected.

**Table 1:** List of checked plants species

Species	Category of IUCN, European and Polish Red Data books	Localization
*Acer pseudoplatanus*	NE	Chojnik, LGB
*Aconitum plicatum*	VU, PRDP,	LGB, MSN, Ł
*Adenostyles alliariae*	NE	MSN
*Alchemilla spp.*	NE	MSN
*Allium sibiricum*	VU, PRDP, ERLVP	LGB, MSN
*Allium victorialis*	EC	MSN
*Anemone alpina*	NE	LBG, MSN, Łabski Szczyt
*Anemone narcissifolia*	NE	MSN
*Arabis alpina*	NE	LGB
*Arnica montana*	VU, PRDP, ERLVP	CZ
*Athyrium distentifolium*	NE	LGB, MSN, WSN, Ł
*Betula carpatica*	NE	Ł
*Campanula bohemica*	EN, PRDP, ERLVP	LGB, MSN
*Cardamine amara*	NE	WSN
*Cardamine resedifolia*	EN, PRDP,	Ł
*Cicerbita alpina*	NE	LBG, WSN, MSN
*Cryptogramma crispa*	CR, PRDP,	WSN
*Daphne mezereum*	NE	MSN
*Digitalis grandiflora*	NE	LBG
*Drosera rotundifolia*	NE	Petrovka
*Empetrum nigrum*	NE	MSN
*Euphrasia minima*	NE	MSN
*Fagus silvatica*	NE	LBG
*Galium sudeticum*	CR, PRDP, ERLVP	LBG, MSN
*Gentianella campestris*	NE	Ł
*Geum montanum*	NE	LBG
*Hieracium spp.*	NE	LBG, MSN
*Hypochaeris uniflora*	NE	LBG
*Lilium martagon*	NE	MSN
*Oxycoccus palustris*	NE	MSN
*Pedicularis sudetica*	EN, PRDP, ERLVP	Ł
*Pinus mugo*	NE	MSN, WSN, CZ, Ł,
*Pleurospermum austriacum*	NE	MSN
*Potentilla aurea*	NE	MSN
*Primula minima*	NE	MSN
*Quercus robur*	NE	LBG, Chojnik
*Ranunculus acris*	NE	MSN
*Ranunculus platanifolius*	NE	MSN
*Rhodiola rosea*	NE	LBG, MSN
*Rosa pendulina*	NE	MSN
*Rubus chamaemorus*	EN, PRDP	Sokolnik
*Saxifraga bryoides*	NE	MSN
*Saxifraga moschata*	EN, PRDP	LBG, MSN
*Saxifraga nivalis*	CR, PRDP	LBG, MSN
*Sedum alpestre*	NE	LBG, MSN
*Sedum maximum*	NE	LBG
*Silene vulgaris*	NE	LBG, MSN
*Solidago alpestris*	NE	LBG
*Sorbus aucuparia*	NE	MSN, Ł
*Swertia perennis*	NE	MSN
*Thesium alpinum*	NE	MSN
*Thymus alpestris*	NE	LBG, MSN
*Vaccinium myrtillus*	NE	WSN
*Vaccinium vitis-idaea*	NE	MSN, WSN, Ł
*Veratrum lobelianum*	NE	MSN, WSN, CZ

*PRD* Polish Red Data Book of Plants (Kaźmierczakowa et al. 2014), *ERLVP* European Red List of Vascular Plants (Bilz et al. 2011), *LBG* Life Bank Gene in Jagniątków, *CZ* Czarny Kocioł, *Ł* Kocioł Łomniczki, *Msn* Mały Śnieżny Kocioł, *Wsn* Wielki Śnieżny Kocioł

The fungi showing sporulation on the surface of infected plants were determined taxonomically in situ. Otherwise, standard phytopathological methods were applied in conducting the mycological analysis of the infested plant organs. Samples of plant tissue showing conspicuous symptoms were taken from infected plants and sliced into *inocula* of ca. 0.2–0.5 cm. Potato dextrose agar (PDA, Biocorp) medium was used for the isolation of fungi that was previously acidified with citric acid (3 mL per 250-mL flask) in order to inhibit the growth of bacteria and for the identification of some species, while Czapek-Dox agar (1.2 % agar, Biocorp) and malt extract agar (MEA, Biocorp) were used for identification of the *Penicillii* and *Aspergilli.* The fungal colonies growing out of the plant stems were transferred and inoculated into slants with PDA medium. After incubation (22 °C, 14–30 days, in darkness), they were subsequently determined to species level based on their morphology, using mycological keys (Raper and Fennell 1965; Raper and Thom 1968; Pitt and Hocking 2009; Watanabe 2011).

## Results

### Field research

**Fig. 2:**
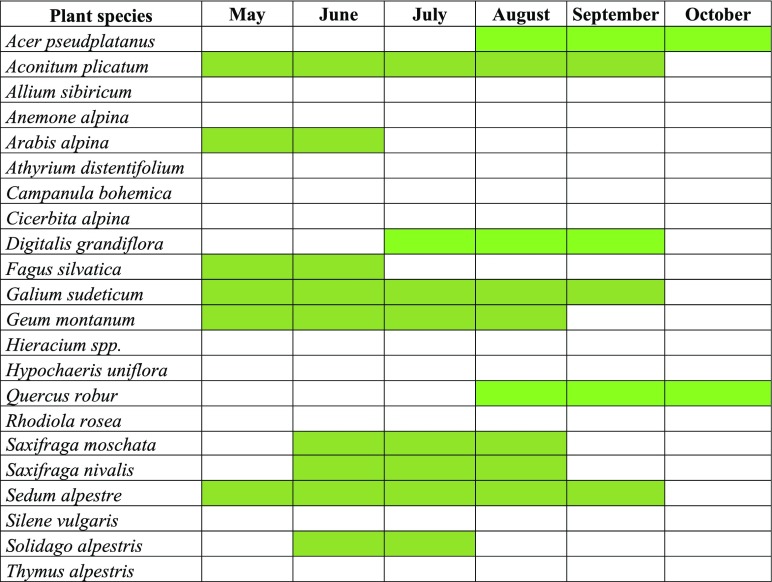
Gantt chart showing incidence of disease symptoms (green) on plants growing in LGB in Jagniątków

**Fig. 3:**
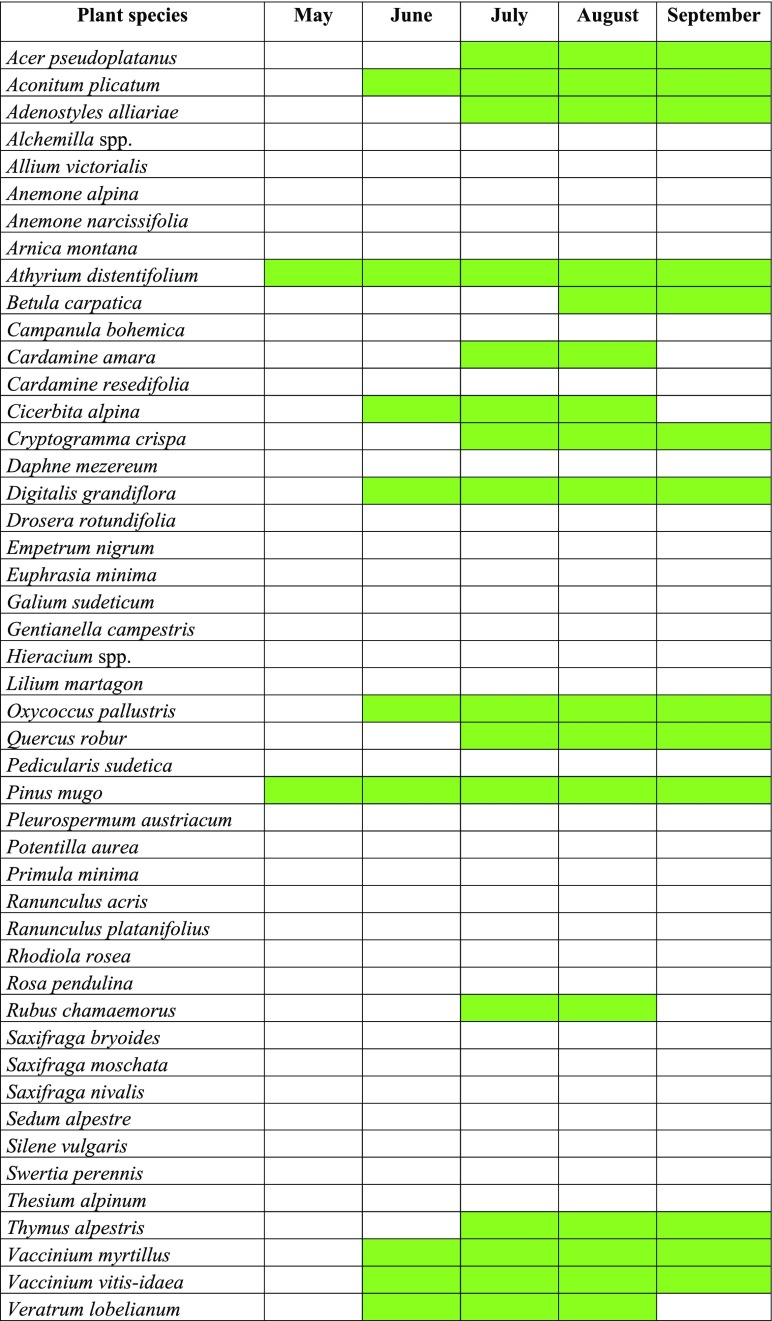
Gantt chart showing incidence of disease symptoms (green) on plants growing in LGB in natural locations in KNP

During the study period, disease symptoms were observed on 30 species of plants. Out of the 21 species monitored within the LGB, 13 (61 %) were determined as diseased (Fig. 2). In turn, among the 47 plant taxa observed within the KNP area, disease symptoms were described on 17 (36 %) (Fig. 3). First symptoms of plant disease in the growth season were observed by the beginning of May in LGB but only at the beginning of June—in natural locations. The greatest intensity of the symptoms occurred at the turn of June and July.

During the second year of the study, the disease symptoms were recorded on the lower number of plants monitored (50 %, 2015), compared to the first year (79 %, 2014). In the natural locations, the symptoms were found on 48 % of plants in the first season (2014) and only on the 34 % in the second (2015) growth season. There is no doubt that the atmospheric conditions prevailing in the second part of the 2015 growth season (high temperatures, drought) have contributed to the situation. Interestingly, some of the observed pathogens, including the culprits of rusts, e.g. *Melampsoridium betulinum*, appeared only later in the season. The similar was true for the LGB in Jagniątków.

### Mycological analysis of the infested plants

The signs of sporulation or incidence of mycelium were detected on 14 species of plants in the course of the field observations (Table 2). The sporulation that was most often observed, testified for the presence of *Uredinales*: *Coleosporium tussilaginis* on *Adenostyles alliariae*, *Melampsoridium betulinum* on *Betula carpactica*, *Puccinia maculosa* on *Cicerbita alpina* or *Phragmidium fusiforme* on *Rosa pendulina*. Moreover, the symptoms brought about by *Erysiphales* were also observed, namely, those of *Sphaerotheca aphanis* on *Geum montanum*, *Erysiphe alphitoides* on *Quercus robur* and *Podospheara alpina* on the leaves of *Saxifraga nivalis* (Table 3).

**Table 2:** The occurrence of fungal pathogen on organs of plants in situ

Plant species	Fungal pathogen
*Acer pseudoplatanus*	*Rhytisma acerinum*
*Adenostyles alliariae*	*Coleosporium tussilaginis*
*Alchemilla* spp.	*Alternaria alternata* *Sclerotinia sclerotiorium*
*Allium sibiricum*	*Peronospora destructans*
*Arabis alpina*	*Sclerotinia sclerotiorium*
*Betula carpatica*	*Melampsoridium betulinum*
*Cicerbita alpina*	*Alternaria alternata* *Puccinia maculosa*
*Digitalis grandiflora*	*Alternaria alternata*
*Hieracium* spp.	*Alternaria alternata* *Cercospora* spp.
*Geum montanum*	*Sphaerotheca aphanis*
*Quercus robur*	*Erysiphe alphitoides*
*Rhodiola rosea*	*Alternaria alternata*
*Rosa pendulina*	*Phragmidium fusiforme*
*Saxifraga nivalis*	*Podosphaera alpina*

**Table 3:** Fungi isolated from infected plants (number of colonies and frequency (%)) in 2014–2015

Fungal species	*Aconitum plicatum*		*Athyrium distentifolium*		*Cardamine amara*		*Cryptogramma crispa*		*Galium sudeticum*		*Pinus mugo*	
Year	2014	2015	2014	2015	2014	2015	2014	2015	2014	2015	2014	2015
*Alternaria alternata*	1 (8.3)	11 (15.1)	12 (50)	1 (2.1)	5 (33.4)	12 (70.6)	1 (7.2)	17 (44.8)	9 (50)	3 (10)		
*Alternaria botrytis*				2 (4.1)						3 (10)		
*Aspergillus* section *Nigri*		3 (4.1)		1 (2.1)			1 (7.2)			1 (3.3)		
*Botrytis cinerea*	5 (41.7)	30 (41.1)					4 (28.6)					
*Chaetomium glacire*	1 (8.3)						4 (28.6)					
*Cladosporium cladosporioides*			1 (4.2)					4 (10.5)			2 (4.7)	
*Cladosporium herbarum*							1 (7.1)					
*Epicoccum nigrum*	1 (8.3)		1 (4.2)		2 (13.4)			4 (10.5)				
*Fusarium culmorum*	1 (8.3)	1 (1.3)	1 (4.2)	21 (42.8)	2 (13.4)	2 (11.8)	3 (21.4)			1 (3.3)		
*Fusarium equiseti*		7 (9.6)		1 (2.1)	1 (6.7)					1 (3.3)		
*Fusarium oxysporum*			5 (20.1)	13 (26.5)	2 (13.4)	3 (17.6)			2 (11.2)	1 (3.3)		
*Fusarium poae*					2 (13.4)							
*Giberella avenacea*			2 (8.3)									
*Lophodermium corconticum*											32 (76.2)	30 (88.3)
*Lophodermium pinastri*											7 (16.7)	2 (5.9)
*Penicillium* sp. 1 section *Chrysogena*				1 (2.1)					1 (5.6)	3 (10)	1 (2.4)	2 (5.9)
*Penicillium* sp. 2 section *Chrysogena*				3 (6.2)				2 (5.3)				
*Pestalotia hartigii*										12 (40)		
*Phoma leveleii*		12 (16.5)		6 (12.2)								
*Rhizoctonia* spp.								3 (7.9)				
*Sclerotinia sclerotiorium*		9 (12.3)	2 (8.3)						3 (16.7)	4 (13.4)		
*Trichoderma harzianum*	3 (25)				1 (6.7)			8 (21.1)	3 (16.7)	1 (3.3)		
*Trichoderma polysporum*								3 (7.9)				
Total numbers of colonies	12	73	24	49	15	17	14	38	18	30	42	34

The mycological analysis yielded detection of 27 species of fungi. In the first year of the study, 22 species were identified, whereas 20 were determined in the next growth season (Tables 4, 5, and 6).

**Table 4:** Fungi isolated from infected plants (number of colonies and frequency (%)) in 2014–2015

Fungal species	*Rubus chamaemorus*		*Salix herbacea*		*Saxifraga moschata*		*Saxifraga nivalis*		*Sedum alpinum*		*Thymus alpestris*	
Year	2014	2015	2014	2015	2014	2015	2014	2015	2014	2015	2014	2015
*Alternaria alternata*	1 (20)	5 (31.2)		19 (86)	4 (12.1)			4 (16.7)		2 (4)		17 (85)
*Alternaria botrytis*		3 (18.7)										3 (15)
*Aspergillus* section *Nigri*			1 (2)							3 (6)		
*Botrytis cinerea*	1 (20)				3 (9.1)					30 (60)		
*Chaetomium glacire*					2 (6.1)							
*Cladosporium cladosporioides*			3 (8)									
*Epicoccum nigrum*					5 (15.1)							
*Fusarium culmorum*	3 (60)							3 (12.5)		1 (2)		
*Fusarium equiseti*			1 (2)									
*Fusarium oxysporum*						6 (18)			5 (33)	1 (2)	1 (100)	
*Fusarium poae*												
*Giberella avenacea*									10 (67)			
*Penicillium* section *Citrina*			9 (26)		3 (9.1)							
*Penicillium* sp. 1 section *Chrysogena*		3 (18.7)	20 (58)				20 (83)	6 (25)				
*Penicillium* sp. 2 section *Chrysogena*										1 (2)		
*Pestalotia hartigii*					1 (3.1)							
*Phoma leveleii*		5 (31.2)		3 (14)	2 (6.1)					12 (12)		
*Sclerotinia sclerotiorium*					6 (18.2)		4 (17)					
*Trichoderma harzianum*					7 (21.2)	21 (63)		9 (37.5)				
*Trichoderma polysporum*						6 (18)		1 (4.2)				
*Trichoderma viridae*								1 (4.2)				
TOTAL numbers of colonies	5	16	34	22	30	33	24	24	15	50	1	20

**Table 5:** Fungi isolated from infected plants (number of colonies and frequency (%)) in 2014 or 2015

Fungal species	*Allium sibiricum*	*Anemone alpina*	*Cardamine resedifolia*	*Vaccinium myrtillus*	*Veratrum lobelianum*	*Arabis alpina*	*Digitalis grandiflora*	*Solidago alpestris*	*Vaccinium oxycoccus*	*Vacciunium vitis-idaea*
Year	2014	2014	2014	2014	2014	2015	2015	2015	2015	2015
*Alternaria alternata*		13 (31.7)	3 (50)	4 (40)	2 (13.4)	3 (25)	6 (24)	30 (90.1)	4 (28.6)	15 (83.4)
*Alternaria botrytis*							1 (4)			
*Arthrinium pheospermum*		7 (17.1)			2 (13.4)					
*Aspergillus* section *Talaromyces*				1 (10)						
*Aspergillus* section *Nigri*										
*Botrytis cinerea*	7 (29.2)	4 (9.7)	1 (16.7)		5 (33.4)					
*Chaetomium glacire*			1 (16.7)		4 (26.7)					
*Cladosporium cladosporioides*		10 (24.4)		1 (10)						
*Epicoccum nigrum*		1 (2.5)	1 (16.7)							
*Fusarium culmorum*							3 (12)	2 (6.1)	6 (42.9)	
*Fusarium oxysporum*		6 (14.7)				2 (16.7)				
*Fusarium poae*										
*Giberella avenacea*	17 (70.8)									
*Penicillium* sp. 2 section *Chrysogena*							1 (4)	1 (3.1)		
*Pestalotia hartigii*					2 (13.4)					
*Phoma leveleii*							11 (44)			
*Sclerotinia sclerotiorium*				4 (40)						3 (16.6)
*Trichoderma harzianum*						7 (58.4)	3 (12)		4 (28.6)	
TOTAL numbers of colonies	24	41	6	10	15	12	25	33	14	18

**Table 6:** Summary juxtaposition of diseases and respective pathogens detected on plants encompassed by the monitoring in 2014–2015

Plant species	Plant diseases	Pathogen	First sypmtoms	Importance
*Acer pseudoplatanus*	Black spot	*Rhytisma acerinum*	VIII	+
*Aconitum plicatum*	Grey mould, leaf and stem spot	*Botrytis cinerea*, *Fusarium* spp.	VI–VIII	++
*Adenostyles alliariae*	Rust	*Coleosporium tussilaginis*	VII–VIII	++
*Alchemilla spp.*	Leaf spot	*Alternaria alternata*	VII	+
*Allium sibiricum*	Downy mildew	*Peronospora destructans*	V	+
*Anemone alpina*	Stem spot	*Alternaria alternata*	VII	++
*Arabis alpina*	White mould	*Sclerotinia sclerotiorum*	V	++
*Athyrium distentifolium*	Leaf and stem spot	*Fusarium* spp., *Giberella* spp.	VI–VII	+++
	Fusarium wilt			
	Leaf’s deformation			
*Betula carpatica*	Rust	*Melampsoridium betulinum*	VII–VIII	+++
*Cardamine amara*	Leaf spot	*Alternaria alternata*, *Fusarium* spp.	VIII–IX	+
*Cardamine resedifolia*	Leaf and stem spot	*Alternaria alternata*	VI	+
*Cicerbita alpina*	Rust	*Puccinia maculosa*	VII	++
*Cryptogramma crispa*	Leaf and stem spot	*Botrytis cinerea*, *Alternaria alternata*	VII–VIII	+++
*Digitalis grandiflora*	Leaf spot	*Alternaria alternata*, *Phoma levellei*	V–VI	+
*Galium sudeticum*	White mould and Fusarium wilt	*Sclerotinia sclerotiorium*, *Fusarium* spp.	V–VI	++
*Geum montanum*	Powdery mildew	*Sphaerotheca aphanis*	IV	++
*Fagus silvaticum*	Root rot, grey mould	*Pythium* spp., *Rhizoctonia* spp., *Botrytis cinerea*, *Fusarium* spp.	V	++
*Hieraclum* spp.	Leaf spot	*Cercospora* spp.	VI	+
*Oxycoccus palustris*	Leaf spot	*Alternaria alternata*, *Fusarium* spp.	VI	++
*Quercus robur*	Powdery mildew	*Erysiphe aplhitoides*	VII	+
*Pinus mugo*	Rust	*Coleosporium tussilaginis*	IV	+
	Brown spot	*Mycosphaerella dearnessi*	IV	++
	Yellow spot	*Lophodermium corconticum*	III	+++
	Needle blight	*Lophodermium pinastri*	IV	+
*Rhodiola rosea*	Leaf and stem spot	*Alternaria alternata*	VI	+
*Rosa pendulina*	Rust	*Phragmidium fusiforme*	VII	+
*Rubus chamaemorus*	Leaf spot	*Alternaria* spp., *Fusarium* spp.	VIII	+++
*Saxifraga moschata*	grey mould, stem spot and Fusarium wilts	*Botrytis cinerea*, *Fusarium* spp.	V–VI	++
*Saxifraga nivalis*	Powdery mildew	*Podosphaera alpina*	VII	++
	White mould and Fusarium wilt	*Sclerotinia sclerotiorium*, *Fusarium* spp.	VII	++
*Sedum alpestre*	Grey mould	*Botrytis cinerea*	V	++
*Solidago alpestris*	Leaf spot	*Alternaria alternata*	VI	+
*Thymus alpestris*	Leaf and stem spot	*Alternaria alternata*, *Fusarium* spp.	V–VI	+
*Vaccinium vitis-idaea*	Leaf spot	*Alternaria alternata*, *Sclerotinia sclerotiorium*	VII	+
*Vaccinium myrtillus*	Leaf spot	*Alternaria alternata*, *Sclerotinia sclerotiorium*	V	+
*Veratrum lobelianum*	Leaf spot	*Alternaria alternata*, *Botrytis cinerea*	VI	+

*+* the disease is not a considerable threat to the monitored plant population; however, systematic monitoring of plant health status and of the extent of disease symptoms is necessary, *++* the disease may become a threat to the monitored plant population; systematic monitoring of plant health status and of the extent of disease symptoms is necessary; on individuals growing in LBG in Jagniątków, fungicide treatments are necessary at the first incidence of disease symptoms, *+++* the disease is a major threat to the monitored plant population; systematic monitoring of plant health status and of the extent of disease symptoms is necessary; on individuals growing in LBG in Jagniątków, fungicide treatments are necessary at the first incidence of disease symptoms

The pathogens isolated most frequently from the infested plant tissues were *Alternaria alternata* (causing leaf and stem spot symptoms) and *Botrytis cinerea* (grey mould) and taxa of the genus *Fusarium* (Fusarium wilt, tissue blight and leaf spot symptoms). From some of the plant species, colonies of the *Penicillium* spp. and *Trichoderma* spp. were isolated as well. The species of these genera are considered as organisms that infest previously infected or already necrosed plant tissues. Among the other pathogenic species found, the following must be mentioned too: of *Sclerotinia sclerotiorium* (white mould), *Phoma levellei* (causing leaf and stem spot symptoms) and *Cladosporium* spp. (leaf spot) (Table 6).

Analysis of the species spectrum of the fungi isolated from the infested plant tissues allows for the conclusion that the atmospheric conditions played an important role in the disease process. The growth season of 2014 was characterized by higher rainfall volume and higher RH of the air compared to 2015, in which the prolonged period of high temperatures occurred, resulting in drought. This latter condition has made the number of the isolated species to decline, in the majority of the cases observed in 2015, although simultaneously, the fungal colony count per tissue sample increased, compared to the 2014 season.

## Discussion

The obtained results suggest indirectly that the phytopathological monitoring, particularly when carried out within areas of high natural value, may prove very useful in many aspects of the broadly understood nature conservation. One of these aspects is the assessment of the health status of rare and endangered plants and, consequently, working out of effective systems for protection and conservation of these species, incorporating such components as timely seed harvesting and storage followed by conservative cultivation of these plants (Alsos et al. 2013). Phytopathological monitoring coupled with the mycological seed analysis may indicate the optimal time for seed harvesting and their further processing. It has been demonstrated in the presented research that the most frequently isolated pathogens are fungi of the genera *Alternaria*, *Fusarium* and *Phoma*, as well as the species of *Botrytis cinerea* and *Sclerotinia sclerotiorium*. These fungi can also infest the seeds of many plant species, including rare and endangered species (Pusz et al. 2016a). Many authors pay attention to the fact that it is the quality of the obtained seed material that is critical for the success in the conservative cultures of rare plant species and, furthermore, for the success of their reintroduction attempts (Cooper et al. 2004; Alsos et al. 2013; Dworzycki and Kroczek 2013). The connection between the harvesting time and seed quality is indisputable, as the factor that negatively affects seed vigour is their moisture content in full maturity (Magan et al. 2010). It therefore seems that, in the conditions of Central Europe, the optimal time to harvest seeds for further plant cultivation, and for creation of conservation archives or seed banks, is the turn of August and September. Weather conditions in Karkonosze Mts. alter drastically already in September, and in most years, the first snowfall is recorded in October or November, thus making seed harvest non-feasible. Moreover, a considerable increase in precipitation is usually recorded still in September. The resulting enhanced RH of the air makes the risk of secodary infections of both plants and seeds still greater (Pusz et al. 2016b).

Another practical aspect of phytopathological monitoring is perhaps the possibility to figure out the changes which occur in time in populations of plants. It seems even more important with respect to plant associations of high natural value, or those which are threatened by extinction. The “symbiotic drift”, the term recognized in ecology, stands for the loss of symbionts by small, isolated and gradually declining populations of hosts, representing changes that are deleterious for such host plant populations (Chlebicki 2004a, b; Chlebicki and Olejniczak 2007). The work of Chlebicki (2004a, b) demonstrates that in populations of *Dryas octopetala* that are numerically large and cover larger swaths of land, there occur considerably more species of (both symbiotic and pathogenic) fungi than in the small, isolated populations of the same plant. This observation conforms to the theory of island biogeography by MacArthur and Wilson (1967). It also seems to be confirmed by the present study, carried out in Karkonosze Mts. The incidence of the six species of fungi was demonstrated on *Rubus chamaemorus* in the years of 2014–2015, among these, both saprotrophic and pathogenic taxa. None of these species is specifically associated to *Rubus chamaemorus.* Interestingly, the number of fungi species that infest organs of the plant is on demise. Chlebicki (1998) had recorded 19 described taxons from all area of *Rubus chamaemorus*, and only 5 of them were cited from Karkonosze Mts., whereas in the next article (Chlebicki 2002), he noted 20 fungal species. After several years, this number has declined to six. The same author had demonstrated that *Rubus chamaemorus* was associated by three species of fungi of boreal-arctic distribution, whereas in the years of 2014–2015, none of them was found on this host. The similar situation was described for *Betula carpatica*. In the course of the monitoring carried out in the present study, only one pathogen species was detected on this plant’s leaves: *Melampsoridium betulinum*. By the end of the nineteenth century, Schroeter had demonstrated the incidence of two species: *Venturia ditricha* and *Gnomonia setacea* (Schroeter 1908). Nearly a century later, Chlebicki (1998) had found seven species there and, interestingly, he did not record among them the species of *Melampsoridium betulinum*. At the same period of time, Chlebicki had been investigating fungi infesting the arctic species of *Saxifraga nivalis*. In the herbary material, he found no fungi species at all. During the present study, in 2014, on one of the individuals of *Saxifraga nivalis* a representative of *Erysiphales* was detected: *Podospheara alpina*. Intriguing as it is, other authors do not report the incidence of this fungus on *S. nivalis*; although three other species recorded from this host plant are listed. These are: *Mycosphaerella densa*, *Mycosphaerella minor*, *Puccinia heucherae* (Chlebicki 1999) and *Wettsteinina douglassi* (Chlebicki 2002).

The above examples illustrate a fluctuating trend in the number of fungi species recorded, as well as the apparent changes in their species composition. They may testify to the changes taking place in these plant populations. Should this phenomenon be associated with the theory of symbiotic drift? Perhaps, yes. Since the work carried out hitherto indicates clearly that the distribution, geographical range and the population size correlate with the number of symbiotic fungi (Chlebicki and Olejniczak 2007), it appears likely that some pathogenic and saprophytic fungi play a role in the process of symbiotic drift too, and this may be especially true for the fungi specialized to infect particular species of host. Interactions between mycorrhizal fungi and pathogens have been investigated in details, but it is still poorly understood what effect these two groups of organisms exert on one another, particularly in extreme habitat conditions, like those prevailing in high mountains (Borowicz 2001; Both et al. 2010). The more important it seems is to determine precisely the effect of pathogens on their host plants, and also on the mycorrhizal fungi, with special attention paid to the endangered species or the species growing in mountainous areas (Both et al. 2010).

Phytopathological monitoring that is carried out permanently, or based on periodically repeated analyses, can lead to the discovery of a species entirely new to science or to a given region. *Lophodermium corconticum*, discovered in Karkonosze Mts., makes a good example (Koukol et al. 2015). This pathogen infects the needles of dwarf mountain pine (*Pinus mugo*), resulting in their premature shedding by the plant (Pusz et al. 2013, Pusz et al. 2016b). It is probably an endemic species and occurs only in Karkonosze Mts. (Koukol et al. 2015, Pusz et al. 2015). In turn, the finding of *Podosphaera alpina* on *Saxifraga nivalis* in 2014 makes the first report on the incidence of this species in Poland. Monitoring that is conducted systematically and may detect an increase or decline of infestation. Such was the case of plant infestations by the recently discovered *Lophodermium corconticum*, the causal agent of the yellow needle blight. The research on the incidence of this disease on dwarf mountain pine, carried out by the author in Karkonosze Mts. in 1998–1999, had shown that at that time, on average, 90 % of the needles bore the symptoms covering up to 25 % of the needle surface area. In the years of 2011–2013, the same symptoms were recorded on 95 % of the needles and they were already covering 70 % of the individual needles’ surface area (Pusz et al. 2013). It points to an apparent progress of disease and may testify to changes in the habitat occupied by dwarf mountain pine, including climate alterations. Meteorological observations conducted in Karkonosze Mts. show changes that manifest themselves with higher temperatures, lower total precipitation, shorter periods with rainfall, lower insolation and lower mean wind velocity in the recent years (2011–2013), compared to the multiannual mean of 1981–2010 (Pusz et al. 2016b).

Climate changes exert considerable influence on all ecosystem elements, including pathogens, as the extent of their incidence is directly dependent on weather conditions in any current season and across the multiannual periods (Yáñez-López et al. 2012). It therefore appears that the observations of the type as described in the present paper have their special merit within cold areas, among them in the high mountains and in polar regions, where the influence of climate alterations is most evident (Ruotsalainen et al. 2004, Pehkonen and Tolvanen 2008).

## Conclusions

The results obtained in the present study justify the statement that the plant healthiness monitoring should become incorporated into the larger system of environmental monitoring. It is of particular importance in regions of high natural value. The 2-year study allowed demonstrating that diseases that occur most often in LGB in Jagniątków and in natural locations in Karkonosze Mts. are leaf and stem spots caused by fungi of the genera *Alternaria* and *Fusarium*, by *Phoma levellei* and by other taxa. Considered the extent of incidence of these diseases and the general condition of plant populations, the systematic monitoring of plant health status is recommended in the natural locations of the following plant species: *Athyrium distenfolium*, *Betula carpatica*, *Cryptogramma crispa*, *Pinus mugo*, *Rubus chamaemorus* and *Saxifraga* spp. This monitoring should be carried out every 2–3 years during the summer months. Seed harvesting for the conservative cultures of plants should be launched at full seed maturity and it should include monitoring of pathogen incidence on seed material. Collecting seeds showing disease symptoms should be avoided. The seeds should be taken along with the entire fruit and stored temporarily in sterile, paper envelopes. Cleaning and drying of the seeds should take place in closed rooms. Seeds should be dealt with using sterile gloves and disinfected tools.
